# Efficacy and safety of tofacitinib in patients with polymyalgia rheumatica: a phase 2 study

**DOI:** 10.1136/ard-2022-223562

**Published:** 2023-01-05

**Authors:** Le Zhang, Jun Li, Hanlin Yin, Dandan Chen, Yuan Li, Liyang Gu, Yakai Fu, Jie Chen, Zhiwei Chen, Shaoying Yang, Shuang Ye, Ting Li, Liangjing Lu

**Affiliations:** 1 Rheumatology, Renji Hospital, Shanghai Jiao Tong University School of Medicine, Shanghai, China; 2 Pharmacy, Renji Hospital, Shanghai Jiao Tong University School of Medicine, Shanghai, China

**Keywords:** Polymyalgia Rheumatica, Glucocorticoids, Therapeutics

Polymyalgia rheumatica (PMR) is an inflammatory disorder characterised by severe pain and stiffness involving the shoulders and proximal aspects of the arms bilaterally. Untreated PMR leads to a significant reduction in quality of life.^1^

Glucocorticoids (GCs) are the mainstay for PMR but induce adverse events in long-term therapy. The clinical need for GC-sparing agents remains unmet. From recent reports, tocilizumab shows efficacy, but the assessment of disease activity might be impaired due to its effect on C reactive protein.^2 3^ For conventional synthetic disease-modifying antirheumatic drugs (csDMARDs), there is no convincing evidence of efficacy. Tofacitinib, a JAK inhibitor, suppresses interferon-γ-related downstream pathway, thereby alleviating the activity of PMR. To explore its efficacy and safety in PMR, we conducted the Simon’s two-stage minimax designed study (NCT04799262).^4^

The key inclusion criteria were patients with highly active PMR (PMR Activity Scale (PMR-AS) >17) with positive inflammatory parameters within 2 weeks prior to screening.^5 6^ The main exclusions included any prior or concurrent use of immunosuppressive therapies within the defined period specified in the protocol, a history of herpes zoster, etc. Tofacitinib of 10 mg/day was administrated concomitantly with prednisone of 15 mg/day at baseline tapered to 2.5 mg/day or less within 20 weeks. GC was tapered following the predefined regimen depending on the response to treatment judged by PMR-AS. During the study, other DMARDs, non-steroidal anti-inflammatory drugs or pregabalin was not allowed. The primary endpoint was the remission response defined as the achievement of sustained low disease activity (LDA) (PMR-AS <7) with GC independence (prednisone ≤2.5 mg/day) for 4 weeks from week 20. Plasma levels of inflammatory cytokines involved in PMR were measured at baseline and endpoint of 24 weeks. The null hypothesis that the true response rate was 0.54 would be tested against a one-sided alternative.^7^ In the first stage, eight patients would be accrued. If there were more than five responses, six additional patients would be recruited for a total of 14. The null hypothesis would be rejected if 11 or more responses were observed in 14 patients. This design yielded a type I error rate of 0.05 and power of 0.8 when the true response rate was 0.85.^2^ Two-sided 95% CIs were calculated to estimate the proportion of responses by the Clopper-Pearson approach. Patients were followed up until 48-week extension. The detailed study procedures are available online (online supplemental file 1).

10.1136/ard-2022-223562.supp1 Supplementary data

From March to December 2021, 14 participants with highly active PMR were enrolled. Eleven patients were newly diagnosed and three were relapsing on prior csDMARDs with an average disease duration of 9.7 (3.9) months. The baseline PMR-AS was 50.9 (25) with a mean Visual Analogue Scale (VAS)-pain of 71.8 (16). At the endpoint, 12 of 14 (85.7%; 95% CI 57.2% to 98.2%) patients reached the remission response (p=0.014) (figure 1A). A significant reduction in PMR-AS and its components was observed at week 2 from baseline and maintained throughout the study; all achieved LDA at week 24 with a median VAS-pain of 5 (0–17.5) at a prednisone dose of 2.2 (1.1) mg/day (table 1). Additionally, the quality of life assessed by Modified Health Assessment Questionnaire and EQ-5D-3L was both significantly improved (p<0.001). A significant decrease was observed in interleukin (IL)-6, tumour necrosis factor-α, BAFF and IL-1Ra (p<0.05) (figure 1C). During the extension, GC-independent LDA persisted without relapse, and GCs and tofacitinib were further tapered according to PMR-AS. At week 48, the average prednisone dose was 1.3 (1.2) mg with six (42.9%) cases of discontinuation; tofacitinib was halved in six subjects while three participants discontinued it. There were 12 adverse events reported by five (35.7%) participants, while no additional cardiovascular disorders, malignancy or herpes zoster infection were recorded (figure 1B).

**Figure 1:**
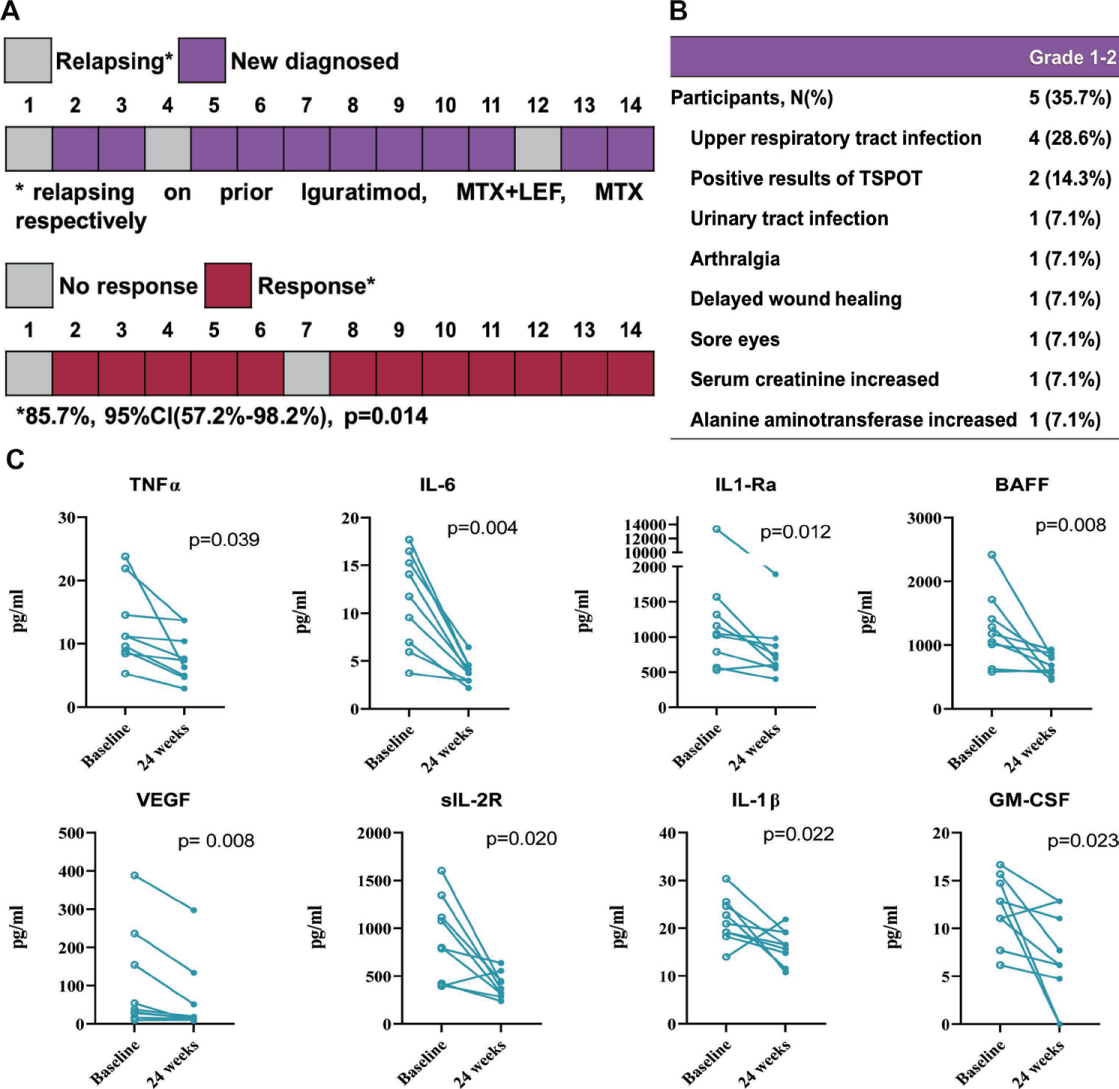
Enrolment and primary outcome (N=14) (A), summary of adverse events (N=14) (B) and changes of inflammatory cytokines in the 24-week study (N=9) (C). GM-CSF, granulocyte-macrophage colony-stimulating factor; IL, interleukin; LEF, leflunomide; MTX, methotrexate; TNF, tumour necrosis factor; VEGF, vascular endothelial growth factor.

**Table 1:** Treatment and disease characteristics during the follow-up (N=14)

	Week 0	Week 2	Week 4	Week 8	Week 12	Week 16	Week 20	Week 24	Week 48
PMR-AS	50.9 (25)	4.0 (2.6–11.3)*	4.3 (3.8)*	4.4 (3.2)*	2.2 (1.1)*	1.3 (0.6–2.8)*	2.2 (1.9)*	2.1 (1.4)*	1.9 (1.5)*
VAS-pain	71.8 (16)	30 (15.6)*	19.3 (14.9)*	11.5 (9.1)*	11.4 (9.9)*	7.9 (7.7)*	2.5 (0–10)*	5 (0–17.5)*	0 (0–0)*
MST (min)	55.9 (14.9)	0 (0–0)*	0 (0–0)*	0 (0)*	0 (0)*	0 (0)*	0 (0)*	0 (0)*	0 (0)*
EUL=0, N (%)	4 (28.6)	12 (85.7)†	14 (100)*	14 (100)*	14 (100)*	14 (100)*	14 (100)*	14 (100)*	14 (100)*
PtGA	7.5 (1.9)	2.5 (1.2)*	1.8 (1.4)*	1.0 (0–2)*	1.1 (1)*	1 (0–1.8)*	0.5 (0–1)*	0.5 (0–1)*	0 (0–1)*
PhGA	7.1 (1.3)	2.5 (1.2)*	1.6 (1.3)*	1.0 (0.9)*	1.1 (0.9)*	1 (0–1)*	0.5 (0–1)*	0 (0–1)*	0 (0–1)*
ESR (mm/hour)‡	66.0 (26.6)	26.9 (19.4)*	10.7 (7)*	15.4 (11.9)*	11.4 (5.7)*	12.3 (10.4)*	13.2 (10.1)*	11.9 (7.4)*	11.4 (7.4)*
CRP (mg/L)	36.5 (26.1)	2 (0.8–9)†	0.9 (0.5–5)†	3.4 (3.2)†	1.1 (0.8)*	0.7 (0.5–1)*	0.7 (0.5–1.5)*	0.8 (0.5–2.5)*	0.5 (0.5–1)*
LDA, N (%)	0	9 (64.3)†	9 (64.3)†	11 (78.6)*	14 (100)*	13 (92.9)*	13 (92.9)*	14 (100)*	14 (100)*
GC (mg/day)	15 (0)	10 (0)*	11.2 (1.2)*	7.9 (1.9)*	5.7 (11.7)*	3.8 (1.2)*	2.2 (0.7)*	2.2 (1.1)*	1.3 (1.2)*
Discontinuation, N (%)	0	0	0	0	0	0	0	0	6 (42.9%)†
Tofacitinib (mg/day)	10 (0)	10 (0)	10 (0)	10 (0)	10 (0)	10 (0)	9.6 (1.3)	9.6 (1.3)	5.7 (3.7)†
Discontinuation, N (%)	0	0	0	0	0	0	0	0	3 (21.4)
MHAQ	3 (1–3)	0.4 (0.2–0.8)*	0.3 (0.1–0.4)*	0.3 (0–0.5)*	0.3 (0–0.3)*	0.1 (0–0.3)*	0.1 (0–0.3)*	0 (0–0.3)*	0 (0–0.2)*
EQ-5D	0.3 (0.2)	0.7 (0.1)*	0.7 (0.1)*	0.8 (0.1)*	0.8 (0.1)*	0.8 (0.1)*	0.9 (0.1)*	0.9 (0.1)*	0.9 (0.1)*

Data are mean (SD) or median (IQR), unless stated otherwise. Significant differences were compared between the visit point and week 0.

*P<0.001.

†P<0.05.

‡The upper limit of normal value of ESR was 20 mm/hour and/or 8 mg/L for CRP.

CRP, C reactive protein; EQ-5D, EuroQol five-dimension questionnaire; ESR, erythrocyte sedimentation rate; EUL, elevation of upper limbs; GC, glucocorticoid; LDA, low disease activity; MHAQ, Modified Health Assessment Questionnaire; MST, morning stiffness; PhGA, Physician’s Global Assessment of VAS for disease activity; PMR-AS, Polymyalgia Rheumatica Activity Scale; PtGA, Patient’s Global Assessment of VAS for disease activity; VAS, Visual Analogue Scale.

To our knowledge, this is the first protocolised study on tofacitinib in PMR. It shows a high clinical efficacy with a good safety profile. Expected benefits may include sustained LDA, improved quality of life and reduced need for GCs.

## Data Availability

The data are available after approval from the corresponding author on reasonable request.
