# Supplementary material for: Efficacy and safety of tofacitinib in patients with polymyalgia rheumatica: a phase 2 study
Source: Ann Rheum Dis. 2023 Jan 5;82(5):722–4. doi: 10.1136/ard-2022-223562 (PMC10176389; doi:10.1136/ard-2022-223562)
Supplement: Supplementary data [file ard-2022-223562supp001.pdf]

Supplement file 1

Study procedures

The trial was conducted following a Simon's two-stage minimax design (*Supplemental figure 1*). After 8 participants completed their 24-week follow up there was an interim analysis. If there were 3 or more failures out of these 8, the trial would stop with the conclusion that the study of tofacitinib should be abandoned. If there were fewer than 3 failures then the study would continue until a further 6 participants received treatment, giving a total sample size of 14. If there were a total of 4 or more failures among 14 participants, it would be concluded that further study should be stopped. If further study of the drug was not abandoned at either the interim or the final analysis, a recommendation to conduct a randomized comparative phase III trial would be made. Patients who completed the 24-week study entered the extension. Efficacy and safety data were presented to week 48 for the intent-to-treat (ITT) participants.

Tofacitinib was administered at a dose of 5 mg twice daily through the 24 weeks. Prednisone of 15 mg daily (or equivalent oral GCs) at baseline was tapered to 2.5 mg or less within 20 weeks. GC would be tapered following the predefined taper regimen depending on the response to treatment judged by PMR-AS based on the shared decision between the patient and the rheumatologist. The PMR-AS was determined every two weeks; if PMR-AS<10 the GC daily dosage would be decreased by 2.5 mg; if PMR-AS>17, the GC daily dosage would be increased to the previous dosage; if 10≤PMR-AS≤17, the GC daily dosage would maintain at the previous stable dose. Any intramuscular, intravenous or intra-articular GCs was not allowed during the study, otherwise the case was considered as treatment failure. Patients who received any intramuscular or intravenous GCs within one week were not eligible to enter this study. Patients who were taking systemic GCs at screening should be on a stable dose≤15 mg/day of prednisone for at least two weeks to be eligible for inclusion in the study. Other DMARDs, nonsteroidal anti-inflammatory drugs (NSAIDs) and pregabalin were not allowed during study, otherwise the case was considered as treatment failure.

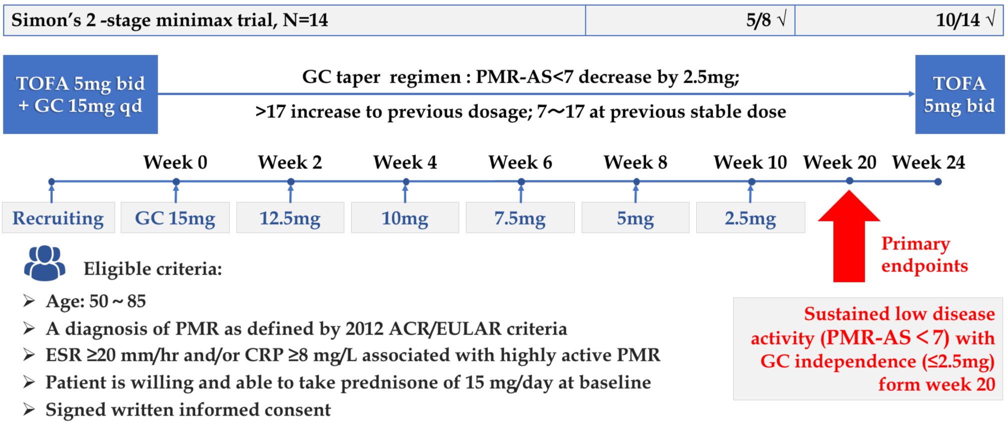

Supplemental figure 1 Study design

TOFA, tofacitinib; GC, glucocorticoid; PMR, polymyalgia rheumatica; PMR-AS, polymyalgia rheumatica activity scale; ESR, erythrocyte sedimentation rate; CRP, C-reactive protein.

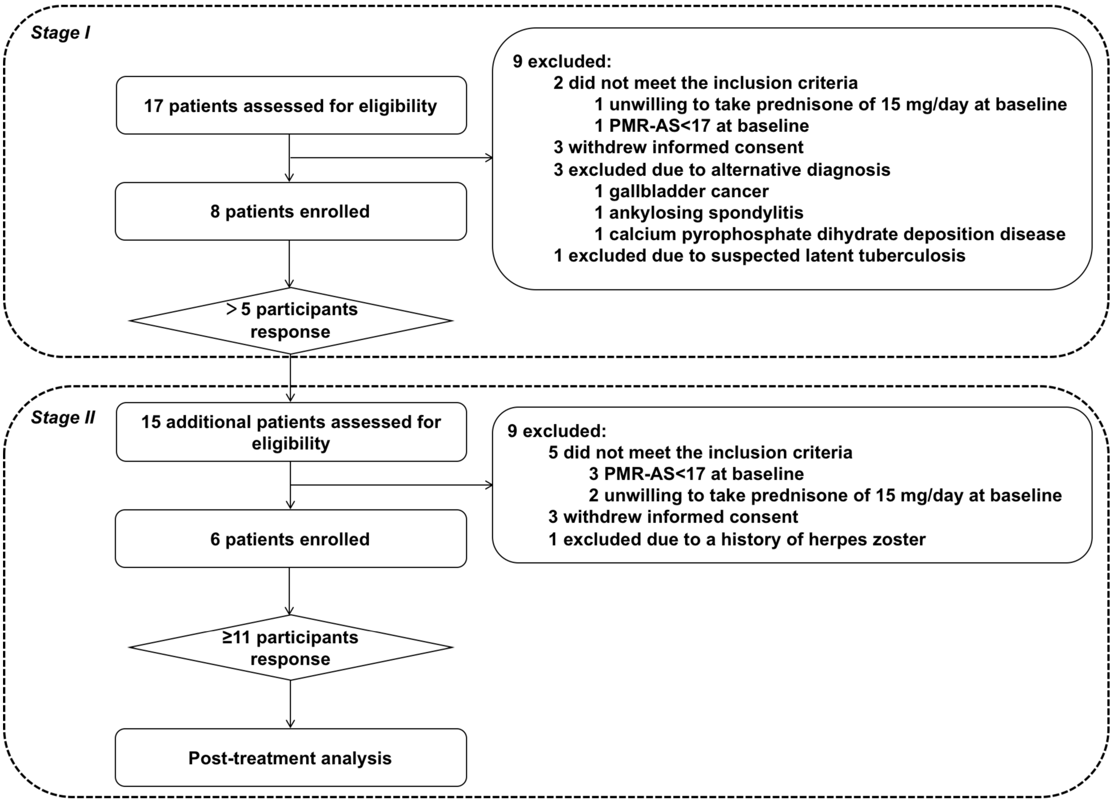

Supplemental figure 2 Study profile

PMR-AS, polymyalgia rheumatica activity scale.

Supplemental table 1 Patient demographics and baseline characteristics

	Tofacitinib (n=14)
Age (years)	69.0 (7.8)
Sex	
Female, N (%)	10 (71.4%)
Body mass index (kg/m^2)	25.1 (3.6)
Relapsing PMR, N (%)	3 (21.4%)
Disease duration, months	9.7 (3.9)
Prior DMARDs, N (%)	
Iguratimod	1/3 (33.3%)
MTX+LEF	1/3 (33.3%)
MTX	1/3 (33.3%)
Comorbidities, N (%)	10 (71.4%)
Cardiovascular diseases	6 (42.9%)
Osteoporosis	4 (28.6%)
Digestive system diseases	4 (28.6%)
Others	3 (21.4%)

Data shown are means (SD), unless stated otherwise. PMR, polymyalgia rheumatica; DMARD, disease-modifying antirheumatic drug; MTX, methotrexate; LEF, leflunomide.

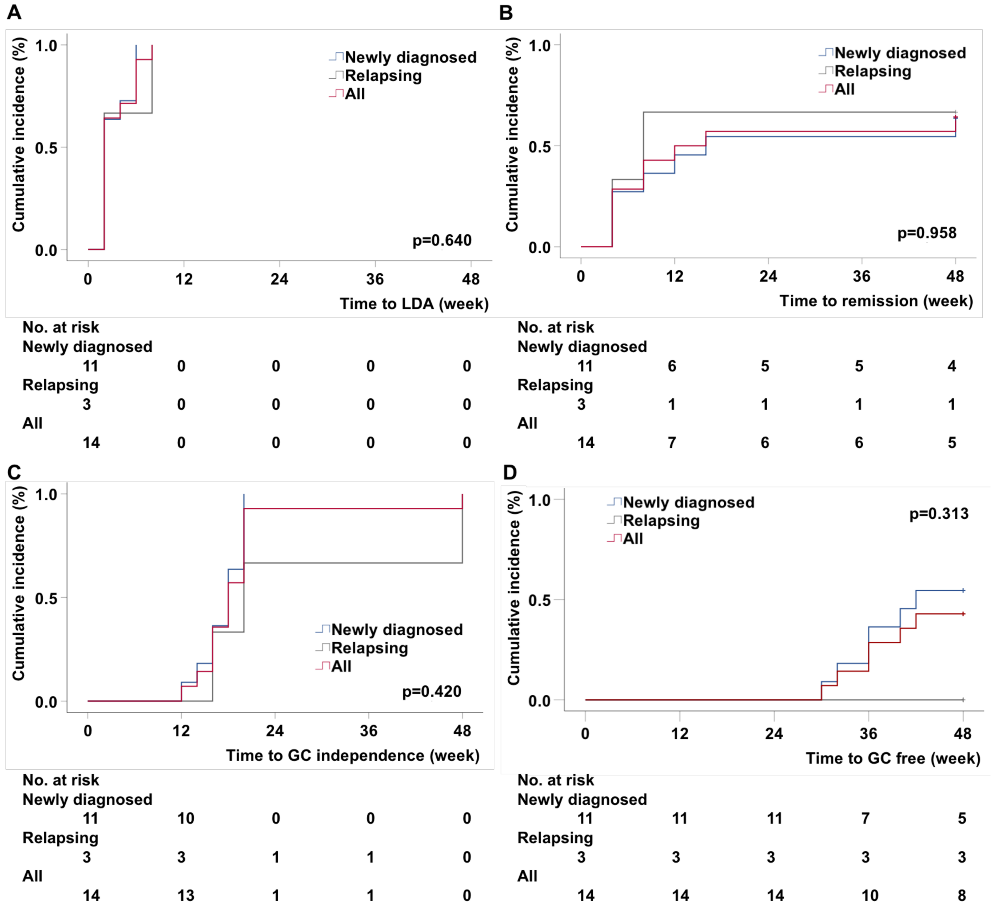

Supplemental figure 3 Kaplan-Meier estimates of LDA (PMR-AS<7) (A), remission (PMR-AS<1.5) (B), GC independence (prednisone≤2.5mg/d) (C) and GC free (D) in the ITT population over 48 weeks. LDA, low disease activity; PMR-AS, PMR activity scale; GC, glucocorticoid.

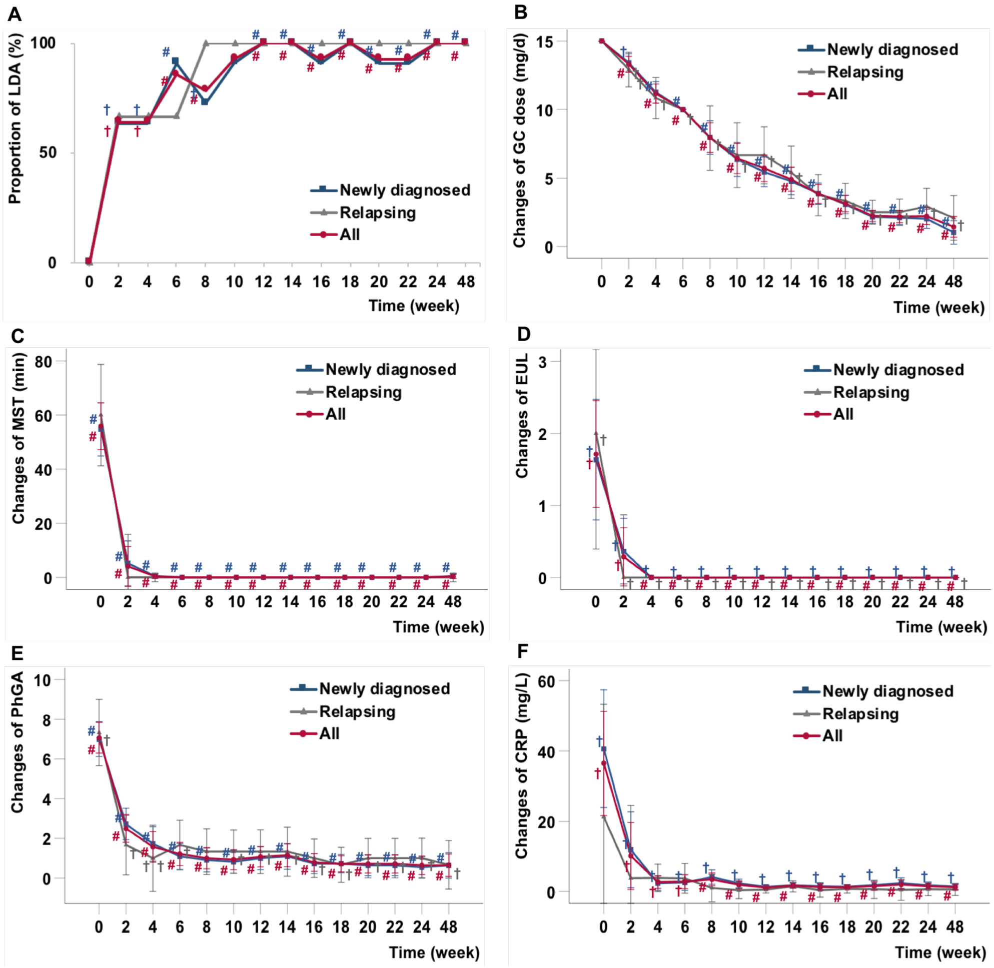

Supplemental figure 4 Increase in proportion of LDA (PMR-AS<7) (A), changes of GC daily dose (B), MST (C), EUL (D), PhGA (E), and CRP (F) over 48-week extension. †p<0.05; #p<0.001.

PMR-AS, polymyalgia rheumatica activity scale; LDA, low disease activity; GC, glucocorticoid; MST, morning stiffness; EUL, elevation of upper limbs; PhGA, physician's global assessment of visual analogue scale for disease activity; CRP, C-reactive protein.
